# Food quality effects on instar‐specific life histories of a holometabolous insect

**DOI:** 10.1002/ece3.5790

**Published:** 2020-01-03

**Authors:** Leslie A. Holmes, William A. Nelson, Stephen C. Lougheed

**Affiliations:** ^1^ Department of Biology Queen's University Kingston ON Canada

**Keywords:** cowpea weevil, juvenile ontogeny, phytophagous consumer, stage‐structured population model

## Abstract

1. It is a long‐standing challenge to understand how changes in food resources impact consumer life history traits and, in turn, impact how organisms interact with their environment. To characterize food quality effects on life history, most studies follow organisms throughout their life cycle and quantify major life events, such as age at maturity or fecundity. From these studies, we know that food quality generally impacts body size, juvenile development, and life span. Importantly, throughout juvenile development, many organisms develop through several stages of growth that can have different interactions with their environment. For example, some parasitoids typically attack larger instars, whereas larval insect predators typically attack smaller instars. Interestingly, most studies lump all juvenile stages together, which ignores these ecological changes over juvenile development.
2. We combine a cross‐sectional experimental approach with a stage‐structured population model to estimate instar‐specific vital rates in the bean weevil, *Callosobruchus maculatus* across a food quality gradient. We characterize food quality effects on the bean weevil's life history traits throughout its juvenile ontogeny to test how food quality impacts instar‐specific vital rates.
3. Vital rates differed across food quality treatments within each instar; however, their effect differed with instar. Weevils consuming low‐quality food spent 38%, 37%, and 18% more time, and were 34%, 53%, and 63% smaller than weevils consuming high‐quality food in the second, third, and fourth instars, respectively. Overall, our results show that consuming poor food quality means slower growth, but that food quality effects on vital rates, growth and development are not equal across instars. Differences in life history traits over juvenile ontogeny in response to food quality may impact how organisms interact with their environment, including how susceptible they are to predation, parasitism, and their competitive ability.

## Graphical abstract

A long‐standing challenge is to understand how changes in host food quality impacts host quality, and how they scale up to influence parasitism rates and parasitoid fitness. Here, we evaluate whether the effects of food quality on host life history are equal throughout ontogeny to ascertain whether trait variation across instars should be considered when characterizing food quality effects. By combining a cross‐sectional experimental approach with a stage‐structured population model to estimate instar‐specific vital rates, we find strong evidence for a trade‐off between host size and development time within an instar suggesting that juvenile instar‐structure is important for host susceptibility to parasitism and parasitoid fitness.

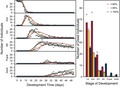

## 1 INTRODUCTION

Food quality in nature varies seasonally and spatially in response to the environment and is often a key determinant of herbivore life history (Awmack & Leather, 2002; Scriber & Slansky, 1981). Organisms can encounter these changes in food quality over the course of their life cycles, which can have consequences for a myriad of traits including body size (Reznick & Yang, 1993), behavior (Bauce, Bidon, & Berthiaume, 2002), and vital rates (e.g., survival, growth, fecundity (Vanni & Lampert, 1992)). These impacts on life history, in turn, can influence how organisms interact with competitors, predators, and resources (Lancaster, Morrison, & Fitt, 2017; Moya‐Larano, 2011; Start, 2018) in their environments.

To understand food quality effects on consumer life histories, most studies follow organisms throughout their life cycles and record major life events, such as age at maturity, in response to changes in food quality (Roff, 1992; Stearns, 1992). From these studies, we know that food quality tends to impact all vital rates. This is well studied in freshwater and marine invertebrate herbivores, where food quality has simultaneous effects on individual consumer growth, development, and reproduction (Sterner & Schulz, 1998 for a review; Vanni & Lampert, 1992 and Walz, 1995 for examples). For example, poor food quality typically results in lower growth rates, smaller body sizes, and reduced fecundity (Cressler, Bengtson, & Nelson, 2017; Sterner & Schulz, 1998). We see similar trends in terrestrial herbivores feeding on host plants (reviewed in Awmack & Leather, 2002). For example, early season or new plant growth is often rich in amino acids, and consumers of these resources typically grow larger and are more fecund than their counterparts feeding on later season or older plants or plant structures (Dixon, 1970; Roslin & Salminen, 2009).

Despite all we have learned from following organisms throughout their life cycles to record major life events in response to food quality, few studies have looked at the effects of food quality during juvenile development (but see Ohmart, Stewart, & Thomas, 1985 and Levesque, Fortin, & Mauffette, 2002 for studies targeting specific juvenile stages of development). Food quality impacts on juvenile development are particularly relevant for organisms with pronounced size‐ or stage‐structure within the juvenile period because different stages can experience different interactions with their environment (Godfray, 1994; Hagstrum & Subramanyam, 2010). For example, susceptibility to parasitism and predation in insects are often instar specific (Benrey & Denno, 1997; Chau & Mackauer, 2000) and therefore larvae in targeted instars may interact with parasitoids and predators, but those in non‐targeted instar stages might not. Competition can also differ across stages, where larger individuals are typically better resource competitors than smaller individuals (i.e., early instars) (Chow & Mackauer, 1984; Van Buskirk, 1992). Similarly, cannibalism commonly occurs between different stages with larger stages cannibalizing smaller stages (Hopper, Crowley, & Kielman, 1996; Pereira, Agostinho, & Winemiller, 2017; Simpson, Joncour, & Nelson, 2018). Food quality studies that lump all juvenile stages together into a single stage miss the details about how food quality impacts the growth, development, and survival among instars and, as a consequence, can miss the impact on important ecological interactions.

The impact of food quality during juvenile development is particularly relevant for arthropods because individuals typically have multiple instars and their interactions with parasitoids, predators, and competitors are typically instar specific (Chau & Mackauer, 2000; Hopper et al., 1996; Van Buskirk, 1992). Outside of the field of forensic entomology, little is known of food quality effects on instar‐specific life histories. For example, in forensic entomology, the instar‐specific growth rates of blowfly larvae (Diptera: Calliphoridae) are used to predict postmortem intervals in medico‐legal investigations (Catts & Goff, 1992; Greenberg, 1991). Among these studies, different types of carrion and tissue types have been shown to impact instar‐specific growth and development (Boatright & Tomberlin, 2010; Wilson, Lafon, Kreitlow, Brewster, & Fell, 2014), suggesting that food quality may impact instar growth and development differently. As a result, we know little about how food quality impacts individual variation in size, development, and survival within and among juvenile instars, where such variation can impact community interactions and food web structure (Moya‐Larano, 2011; Start, 2018).

Here, we study the developmental rates, growth, and survival of juvenile bean weevils for each larval instar in response to manipulations of food quality to test whether the effects of food quality on larval life history traits are equal throughout juvenile ontogeny. We use the bean weevil *Callosobruchus maculatus* (Fabricius) (Coleoptera: Bruchidae) because it is an herbaceous pest of seed pulses that is easily maintained in the laboratory on both natural and artificial seed pulses. We destructively sample individual bean weevils throughout their entire juvenile ontogeny to estimate instar‐specific growth and survival rates across a food quality gradient using a cross‐sectional experimental approach combined with a stage‐structured population model. Importantly, by tracking both the number of live and dead animals on each sampling date, we can estimate both instar‐specific development and mortality rates from the observed time series (Nelson, McCauley, & Wimbert, 2004) and show that food quality effects on larval life histories vary significantly across instars.

## 2 MATERIALS AND METHODS

### 2.1 Callosobruchus maculatus life‐history

Stock populations of *C. maculatus* were reared on black‐eye peas *Vigna unguiculata* and maintained in a Conviron CMP 3,244 climate‐controlled growth chamber (Controlled Environments Ltd.) at 28°C, 75% relative humidity, and a 12:12 [L:D] fluorescent light regime. Adult *C. maculatus* lay eggs on the surface of seed pulses. Upon hatching, first instar larvae burrow into the pulse, feeding on the inner flesh. Larvae develop through four instars, each time molting their exoskeleton and head capsule (Larson, 1938). At the end of their fourth instar, larvae will molt a final time and enter the pupal stage, after which they metamorphose. Adults are sexually mature at eclosion and can immediately mate and reproduce. Development from egg to adult takes approximately 28 days in the above laboratory conditions (L. A. Holmes, unpublished data).

### 2.2 Artificial black‐eye peas

The traditional approach to manipulating food quality is to use pulses of different species (e.g., Kazemi, Talebi, Fathipour, & Farahani, 2009; Messina, 2004). However, this confounds food quality with plant physiology, plant defense compounds, and overall pulse size. To control for these confounds, we created artificial pulses from black‐eye pea flour (hereafter called pellets). We created pellets varying in quality by adding different amounts of indigestible crude fiber and lignin (filler) from milled peanut shells to ground black‐eye pea (flour) in the following proportions: 90:10; 95:5; and 100:0 of flour:filler as % dry mass.

### 2.3 Characterizing resource quality effects on weevil life history

In preparation for our experiments, mated colony stock *C. maculatus* females were isolated and placed individually in 24‐well culture plates that contained a single black‐eye pea pulse in each plate well. Females were allowed to oviposit for 8 hr, after which they were removed from their pulse. The eggs on each pulse were thus from a single female. Pulses were placed in the same growth chamber as the stock colony. After a 4‐day incubation period, a single, viable egg (identified by the presence of a developing head capsule) was removed from each pulse using a dissecting microscope and a scalpel, ensuring that each egg in the experiment originated from a separate female. Eggs were then transferred to individual artificial pellets and returned to the growth chamber.

Immature weevils cannot survive outside of their pellet; thus, we used destructive sampling to quantify the effects of food quality on weevils' daily growth, development, and survival. Four‐day‐old eggs collected from stock *C. maculatus* females were transferred to pellets for the three pellet quality treatments (100%, 95%, and 90% black‐eye pea flour). Beginning on day 5 of the experiment, (i.e., 5‐day‐old weevils), a single pellet was removed each day for 45 days (i.e., 50 days of weevil development from hatching) from each treatment in each replicate. Sampled pellets were placed in a −10°C freezer to stop growth and development of the larva, giving us cross‐sectional data on development and survival every 24 hr. We retrieved the larvae from each frozen pellet using a scalpel under a dissecting microscope. The experiment was replicated 40 times, such that the total number of sampled larvae (40 replicates × 46 days × 3 treatments) was 5,520.

For each dissected pellet, we noted survivorship (i.e., whether an individual survived until its freeze date), identified larval instar by counting the number of head capsule molts recovered during dissection, measured larval head capsule width at its largest width using 6X magnification and ocular micrometer, and quantified larval dry mass using a microbalance scale (Mettler Toledo XP6, Canada). Individuals that were discolored (e.g., brown, black or yellow in color) and/or appeared to be desiccated (hard and impenetrable) were considered to have died prior to being frozen and were characterized as being dead before their sampling date. Samples were dried in a drying oven at 76**°**C for 72 hr. Dry mass was measured three times, and the average of these three measurements was used for all subsequent analysis.

### 2.4 Testing for differences in morphology

All statistical analyses were done using the R software environment version 3.4.4 (R Core Team, 2018).

#### 2.4.1 Head capsule width distribution across pellet quality

We used a generalized linear model to characterize the change in weevil head capsule width over larval instars for each food quality treatment. Preliminary data exploration indicated a positive mean–variance relationship in the residuals, which was accommodated using an overdispersed Poisson distribution. The statistical model was

Systematic:Y∼Q∗S

Error:quasi Poisson with aloglink

where *Y* is head capsule width, *Q* is pellet quality treatment, and *S* is observed larval instar (*L*1, *L*2, *L*3, and *L*4). Statistical significance was evaluated using model selection (Burnham & Anderson, 2002) with quasi Akaike Information Criteria (qAIC). The values for qAIC were calculated using the *bbmle* package (Bolker & R Development Core Team, 2017). To test for an interaction between pellet quality and stage of development on head capsule widths, we used the general linear hypothesis testing function *glht* in the *Multcomp* package (Hothorn, Bretz, & Westfall, 2008).

#### 2.4.2 Body size distribution across pellet quality

Dry mass was used to characterize the distribution of weevil biomass throughout juvenile development for each pellet quality treatment. Preliminary data exploration revealed a strong mean–variance relationship that was well characterized by an overdispersed Gamma distribution. The statistical model was

Systematic:Y∼Q∗S

Error:quasi Gamma with an inverse link

where *Y* is head capsule width, *Q* is pellet quality treatment, and *S* is observed larval instar (*L*1, *L*2, *L*3, and *L*4). Statistical analysis followed the same steps as for head capsule width.

### 2.5 Inferring stage‐specific development and mortality rates

The need for destructive sampling creates two challenges. The first is that life‐history rates (stage‐specific development and mortality) must be inferred from time‐series data on the number individuals in each stage through time (Wood, 1994). Second, while we can observe the stage of an individual at the time of sampling, and whether it is alive or dead, it is not possible to know its age at death. Thus, the resulting data are the numbers of individuals in a particular stage through time and the cumulative number that have died in each stage. Life‐history rates can be inferred from such data by fitting a stage‐structured model to the time‐series data (Wood, 1994) and can be used to infer both stage‐specific development and stage‐specific mortality rates (Nelson et al., 2004).

The population model is a generic stage‐structure model with six stages; five juvenile stages (*L*1, *L*2, *L*3, *L*4, and *L*5) and one adult stage (*A*). Per‐capita mortality rates and the distribution of development times are assumed to be constant over time within a stage but are stage‐specific. Recruitment is known and controlled experimentally. To estimate both the mean and the variation in development time across stages and pellet quality treatments, we used a distributed‐delay model based on Gamma distributions of development time (Bjørnstad, Nelson, & Tobin, 2016). The formalism works by dividing each stage into *k* substages with a constant stage‐specific development rate through each. Depending on the number of substages, the distribution of development times can fall between an exponential distribution (*k* = 1) and a Dirac distribution (*k* = ∞), which means that the model can accommodate a wide range of development distributions. The resulting distributed‐delay model was solved with the following set of ordinary differential equations:

(1) $$\begin{array}{c} \frac{dL_{1,j}\left(t\right)}{\mathrm{dt}}=\left\{\begin{array}{cc} -\left(k_{1}\alpha_{1}+\delta_{1}\right)L_{1,1}\left(t\right) & \text{if}\;j=1\\ K_{1}\alpha_{1}L_{1,j-1}\left(t\right)-\left(k_{1}\alpha_{1}+\delta_{1}\right)L_{1,j}\left(t\right) & \text{if}\;j>1 \end{array}\right.\\ \frac{dL_{2,j}\left(t\right)}{\mathrm{dt}}=\left\{\begin{array}{cc} K_{1}\alpha_{1}L_{1,k_{1}}\left(t\right)-\left(k_{2}\alpha_{2}+\delta_{2}\right)L_{2,1}\left(t\right) & \text{if}\;j=1\\ K_{2}\alpha_{2}L_{2,j-1}\left(t\right)-\left(k_{2}\alpha_{2}+\delta_{2}\right)L_{2,j}\left(t\right) & \text{if}\;j>1 \end{array}\right.\\ \frac{dL_{3,j}\left(t\right)}{\mathrm{dt}}=\left\{\begin{array}{cc} K_{2}\alpha_{2}L_{2,k_{2}}\left(t\right)-\left(k_{3}\alpha_{3}+\delta_{3}\right)L_{3,1}\left(t\right) & \text{if}\;j=1\\ K_{3}\alpha_{3}L_{3,j-1}\left(t\right)-\left(k_{3}\alpha_{3}+\delta_{3}\right)L_{3,j}\left(t\right) & \text{if}\;j>1 \end{array}\right.\\ \frac{dL_{4,j}\left(t\right)}{\mathrm{dt}}=\left\{\begin{array}{cc} K_{3}\alpha_{3}L_{3,k_{3}}\left(t\right)-\left(k_{4}\alpha_{4}+\delta_{4}\right)L_{4,1}\left(t\right) & \text{if}\;j=1\\ K_{4}\alpha_{4}L_{4,j-1}\left(t\right)-\left(k_{4}\alpha_{4}+\delta_{4}\right)L_{4,j}\left(t\right) & \text{if}\;j>1 \end{array}\right.\\ \frac{dL_{5,j}\left(t\right)}{\mathrm{dt}}=\left\{\begin{array}{cc} K_{4}\alpha_{4}L_{4,k_{4}}\left(t\right)-\left(k_{5}\alpha_{5}+\delta_{5}\right)L_{5,1}\left(t\right) & \text{if}\;j=1\\ K_{5}\alpha_{5}L_{5,j-1}\left(t\right)-\left(k_{5}\alpha_{5}+\delta_{5}\right)L_{5,j}\left(t\right) & \text{if}\;j>1 \end{array}\right.\\ \frac{dA(t)}{\mathrm{dt}}=k_{5}\alpha_{5}L_{5,k_{5}}\left(t\right)-\delta_{A}A\left(t\right)\\ L_{i}\left(t\right)=\sum_{j=1}^{\mathrm{ki}}L_{i,j}\left(t\right) \end{array}$$

where *L_i,j_*(*t*) is the number of larvae of stage *i* (*L*1, *L*2, *L*3, *L*4, and *L*5) in substage *j* at time *t* and *A_j_*(*t*) is the number of adults in substage *j* at time *t*. The total number of individuals in each stage is *L_i_*(*t*) and *A*(*t*) for larvae and adults, respectively, which are derived by fitting the state variables to the data. Per‐capita mortality rate for the *i*th larval stage is *δ_i_* and for the adult stage is *δ_A_*. Development rate for the *i*th larval stage is *α_i_*, and the number of substages is given by *k_i_* for larvae. These ordinary differential equations were solved with the experimental conditions of *L*_1_(0) = 40, *L_i,j_(0)*, *i* > 0 = 0, *A_j_*(0) = 0.

Our goal was to estimate the stage‐specific mortality and development rates for each pellet quality treatment. In terms of the model, this corresponds to estimating the *δ* and *α* parameters for each stage and pellet quality treatment. Following Nelson et al. (2004), this requires combining likelihood values from both the time series of stage abundances of live animals and the cumulative number of individuals that die from each stage for each pellet quality. We created an objective function (*f*(*δ*, *α*, *k*)) (Equation 2) that assumes a Poisson error distribution to compare the predicted number of individual weevils from the stage‐structured population model with the observed data:

(2) $$\begin{array}{c} f\left(\hat{\delta },\hat{\alpha },\hat{k}\right)=-\sum_{i=1}^{n}\sum_{j=1}^{m}2\left(N_{L_{\mathrm{ij}}}\log\left(\frac{N_{L_{\mathrm{ij}}}}{{\tilde{N}}_{L_{\mathrm{ij}}}}\right)-\left(N_{L_{\mathrm{ij}}}-{\tilde{N}}_{L_{\mathrm{ij}}}\right)\right)\\ -\lambda \left(N_{D_{\mathrm{ij}}}\log\left(\frac{N_{D_{\mathrm{ij}}}}{{\tilde{N}}_{D_{\mathrm{ij}}}}\right)-\left(N_{D_{\mathrm{ij}}}-{\tilde{N}}_{D_{\mathrm{ij}}}\right)\right) \end{array}$$

where *j* = (1, 2, …, *m*) is the index for the observations, *i* = (1, 2, …, *n*) is the index for stages, $N_{L_{\mathrm{ij}}}$ is the *j*th observation of the number of living individuals in the *i*th stage, and ${\tilde{N}}_{L_{\mathrm{ij}}}$ is the *j*th prediction of the number of living individuals in the *i*th stage. $N_{D_{\mathrm{ij}}}$ is the *j*th observation of the number of dead individuals in the *i*th stage, and ${\tilde{N}}_{D_{\mathrm{ij}}}$ is the *j*th prediction of the number of dead individuals in the *i*th stage. The parameter lambda, λ, is a weighting factor to correct for the imbalance in the number of living and dead observations because the number of living observations recorded per stage per day is greater than the cumulative number of dead observations per stage. Lambda was weighted as a ratio of the sum of *N_L_* to the sum of *N_D_* for all stages. Using the optimization routine, *nmkb* from the package *dfoptim* (Varadhan, Borchers, & ABB Corporate Research, 2017) that implements the Nelder‐Mead algorithm for optimization with parameter bounds, we estimated the most likely values for each parameter, *δ*, *α*, and *k* for each pellet quality that would minimize the objective function. Since the parameters being optimized refer to developmental and mortality rates, it was necessary to set biologically realistic bounds on the parameters. We set an upper (*δ* = 0.1, *α* = 1, and *k* = 120) and lower (*δ* = 0.0001, *α* = 0.01, and *k* = 1) bound for each parameter, respectively.

Confidence intervals for weevil development times (*α* ^−1^) and mortality rates (*δ*) were estimated from 30,000 parametric bootstraps. The residuals showed substantial over or under dispersion depending on stage (Table S1). To account for the mean–variance relationship in the residuals, we generated Tweedie random deviates using the *rTweedie* function from the package *mgcv* (Wood, 2017), where the value of the scale parameter was estimated from the observed indices of dispersion (ESM, Table S1). We did not consider any autocorrelation of residuals because the destructive sampling design of the experiment means that the observations were independent once the population dynamics were characterized. Survivorship (*S_i_*) through each stage of development *i* (*L*1, *L*2, *L*3, *L*4, and *L*5) was estimated using the most likely parameter values, *α* ^−1^ and *δ* as $S_{i}=e^{-\frac{\delta_{i}}{\alpha_{i}}}$.

Statistical evaluation of the differences in development time and survivorship was done using nonparametric bootstrapping. Stage‐specific null distributions of weevil development times and survivorships were estimated by resampling all individuals that were alive in a particular stage through time and the cumulative number of individuals that had died in a stage without regard to their treatment. The most likely value for each parameter (*δ*, *α*, and *k*) was estimated for each resampled dataset by solving the same set of ordinary differential equations (Equation 1), using the same objective function (Equation 2) and optimization routine described above. Resampling was done 15,000 times resulting in a bootstrapped distribution of parameter estimates for each stage, which were then used to create the stage‐specific null distributions of differences for development time (*α* ^−1^) and survivorship (*S*). The probability of observing differences in the most likely estimates of *α* and *S* among pellet quality levels was calculated for each stage of development from the stage‐specific null distributions of differences of each parameter using the percentile method following Efron (1982).

## 3 RESULTS

**Figure 1:**
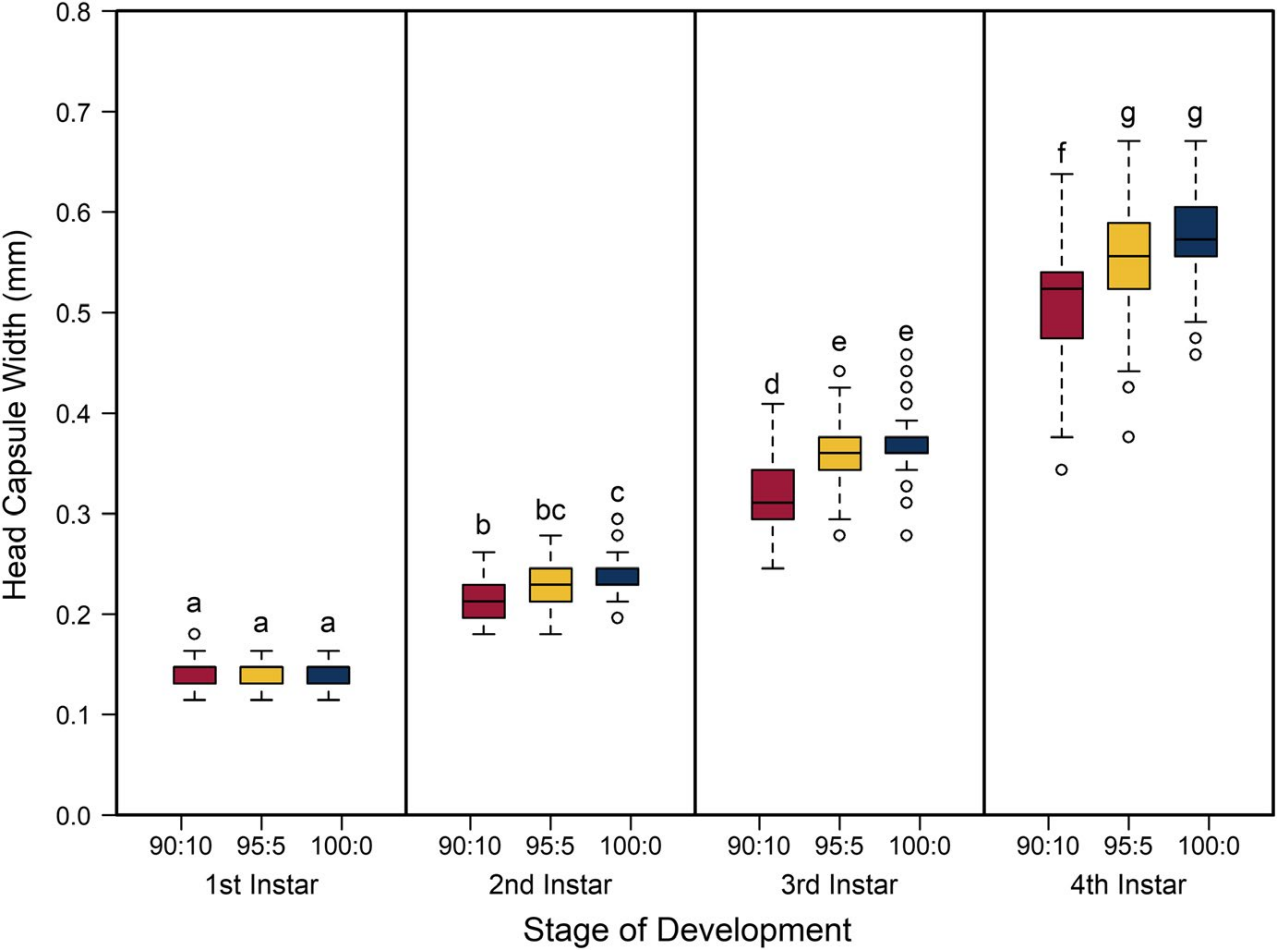
Boxplots of head capsule widths (mm) of *Callosobruchus maculatus* measured across juvenile stages of development (1st, 2nd, 3rd, and 4th larval instars) for each pellet quality treatment (90% black‐eye pea: 10% filler, 95% black‐eye pea: 5% filler, and 100% black‐eye pea: 0% filler, represented by red, yellow, and blue, respectively). The horizonal line within the box indicates the median, the lower, and the upper boundaries of the box indicate the 25th and 75th percentiles, respectively, and the upper and lower whiskers (dashed lines protruding from each box) represent largest and smallest nonoutlier head capsule widths, respectively. Points above and below each whisker represent potential outliers, as they are more than 1.5 times either the upper or the lower boundary, respectively. The full model with an interaction term between pellet quality and juvenile stage of development computed the lowest qAIC (qAIC = 137,045.4), indicating that the interaction between pellet quality and juvenile stage best models the distribution of head capsule width. Head capsule width generally increased with pellet quality and juvenile stage, beginning in larval instar 2. Boxplots with different letters are significantly different (*p* < .01)

**Figure 2:**
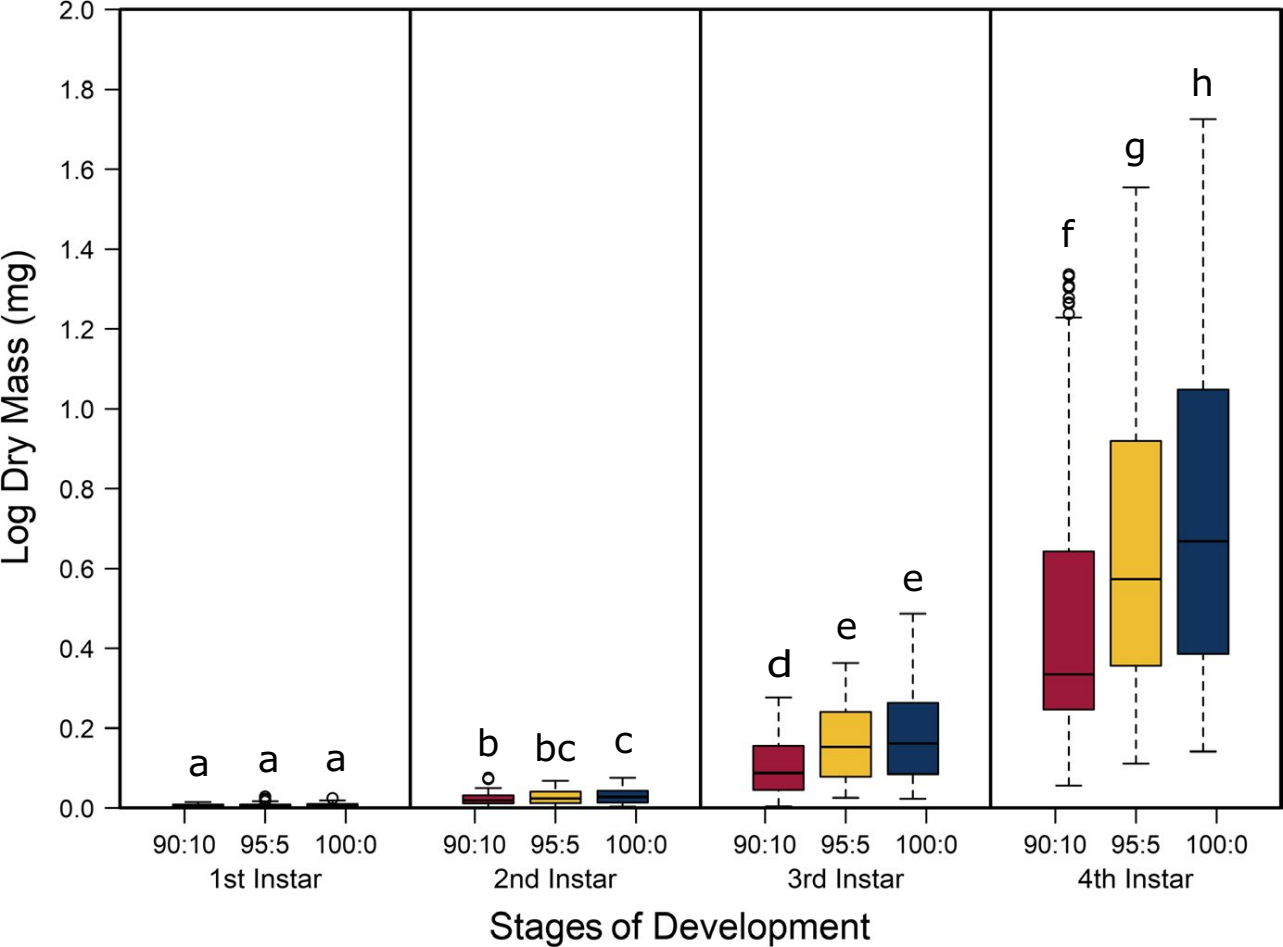
Boxplots of log dry biomass of *Callosobruchus maculatus* measured in milligrams across juvenile stages of development (1st, 2nd, 3rd, and 4th larval instars) for each pellet quality treatment (90% black‐eye pea: 10% filler, 95% black‐eye pea: 5% filler, and 100% black‐eye pea: 0% filler, represented by red, yellow, and blue, respectively). Other details are as in Figure 1. The full model with an interaction term between pellet quality and juvenile stage of development computed the lowest qAIC (qAIC = 137,045.4), indicating that the interaction between pellet quality and juvenile stage best models the distribution of dry biomass. Dry biomass generally increased with pellet quality and juvenile stage, beginning in larval instar 2, but differences in dry biomass among 95% black‐eye pea: 5% filler and 100% black‐eye pea: 0% filler are not apparent until the fourth larval instar. Boxplots with different letters are significantly different (*p* < .01)

The best model for predicting both head capsule width and dry biomass of *C. maculatus* included the interaction between larval stage of development (*S*) and pellet quality treatment (*Q*) (Tables 1 and 2, respectively). Overall, head capsule width and biomass increased with instar and pellet quality, but patterns of increase across pellet quality treatments within an instar were not evident until the second instar (Figures 1 and 2, respectively; ESM: Tables S2 and S3, respectively). Specifically, there were no differences in mean head capsule width or mean dry biomass in the first larval instar across pellet quality treatments, but in the second and subsequent instar stages, mean head capsule width and mean dry biomass generally increased with pellet quality. Moreover, patterns of increase in head capsule width and biomass were similar until the fourth instar. Specifically, differences in both head capsule width and biomass were found between 90% and 100% pellet quality treatments in the second and subsequent instar stages of development. Similarly, head capsule width and dry biomass were significantly different between 90% and 95% pellet quality treatments in the third and fourth instars, but we found no differences in head capsule width between 95% and 100% in any stage, and differences in dry biomass between 95% and 100% were only seen in the fourth instar (Figures 1 and 2, respectively; ESM: Tables S2 and S3, respectively).

**Table 1:** Model selection for the effect of both pellet quality treatment (*Q*) (90% black‐eye pea: 10% filler, 95% black‐eye pea: 5% filler, and 100% black‐eye pea: 0% filler) and developmental stage (*S*) (*L*1, *L*2, *L*3, and *L*4) on head capsule width of *Callosobruchus maculatus* fit to 4,349 observations with 25,347.8 null deviance

Model	ΔqAIC	*df*	Weight	Residual deviance
*Y* ~ *Q* * *S*	0.0	12	1	680.1
*Y* ~ *Q* + *S*	223.2	6	<0.001	911.0
*Y* ~ *S*	1,471.0	4	<0.001	716.6
*Y* ~ *Q*	155,878.4	3	<0.001	24,890.8
*Y* ~ 1	158,870.7	1	<0.001	25,346.8

**Table 2:** Model selection for the effect of both pellet quality treatment (*Q*) (90% black‐eye pea: 10% filler, 95% black‐eye pea: 5% filler, and 100% black‐eye pea: 0% filler) and developmental stage (*S*) (*L*1, *L*2, *L*3, and *L*4) on dry biomass of *Callosobruchus maculatus* fit to 3,502 observations with 10,463.2 null deviance

Model	ΔqAIC	*df*	Weight	Residual deviance
*Y* ~ *Q* * *S*	0.0	13	1	1,938.7
*Y* ~ *Q* + *S*	121.7	7	<0.001	1,974.9
*Y* ~ *S*	580.1	5	<0.001	2,105.0
*Y* ~ *Q*	580.1	5	<0.001	10,071.9
*Y* ~ 1	13,406.2	2	<0.001	10,463.2

**Figure 3:**
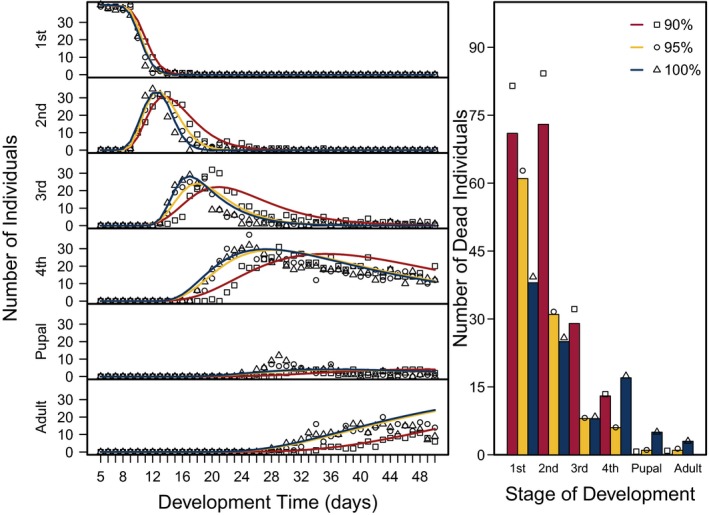
Fitted stage‐structured model to time‐series data comprising the number of *Callosobruchus maculatus* in a particular stage of development (1st, 2nd, 3rd, and 4th instar, pupal and adult) through time (left), and the cumulative number of *C. maculatus* that died in each stage of development (right) for each pellet quality (90% black‐eye pea: 10% filler, 95% black‐eye pea: 5% filler, and 100% black‐eye pea: 0% filler). Left: observed (symbols) and predicted (lines) number of living *C. maculatus* individuals recorded daily for each stage of development and pellet quality treatment. Predicted number of *C. maculatus* were estimated by solving a distributed‐delay model with a set of ordinary differential equations using an objective function that assumes a Poisson error distribution that compared the predicted number of individual weevils from the stage‐structured population model to the observed data and calculated the likelihood of the distribution as a function of the initial parameters. Right: observed (bars) and predicted (symbols) number of dead *C. maculatus* individuals recorded for each stage of development and pellet quality treatment

**Figure 4:**
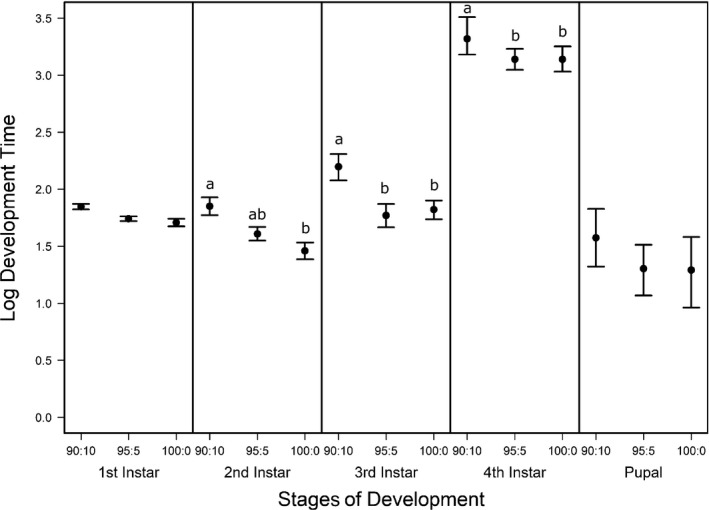
Mean and 95% confidence intervals of 30,000 bootstrapped parameter estimates of *Callosobruchus maculatus* development time for three pellet qualities (90% black‐eye pea: 10% filler, 95% black‐eye pea: 5% filler, and 100% black‐eye pea: 0% filler) in each stage of weevil development (1st, 2nd, 3rd, and 4th instars, pupal and adult). The distribution of 30,000 bootstrapped parameter estimates was computed by fitting a stage‐structured model to time‐series data that comprised resampling the number of *C. maculatus* in a particular stage of development (1st, 2nd, 3rd, and 4th instar, pupal and adult) through time, and the cumulative number of *C. maculatus* that died in each stage of development. To infer significance of pellet quality differences on development time of *C. maculatus* within each stage of development, development time differences across pellet qualities estimated from the observed data were compared with null distributions of differences of resampled development times for each pellet quality comparison (90%–95%, 95%–100%, and 90%–100% black‐eye pea flour). Pellet quality comparisons of *C. maculatus* development time labeled with different letters within each stage of weevil development, are significantly different from the null difference of resampled *C. maculatus* development times, (*p* < .05)

**Figure 5:**
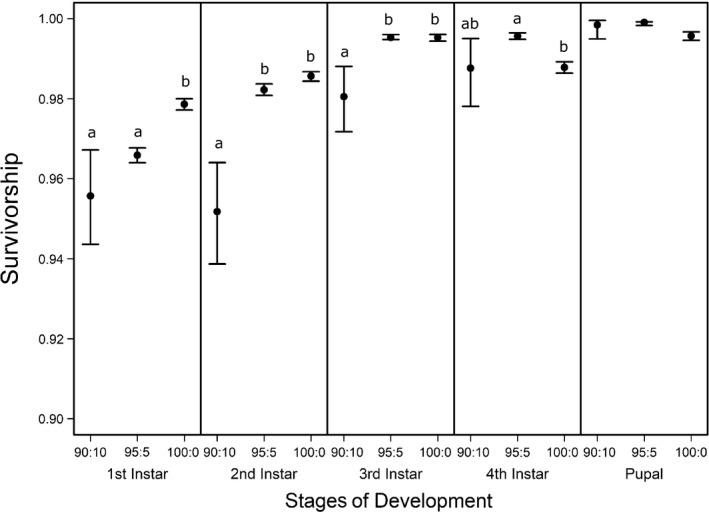
Mean and 95% confidence intervals of survivorship ($e^{-\delta \alpha^{-1}}$) of *C. maculatus* through each stage of development (1st, 2nd, 3rd, and 4th instars, and pupal) for three pellet qualities (90% black‐eye pea: 10% filler, 95% black‐eye pea: 5% filler, and 100% black‐eye pea: 0% filler). Survivorship means and 95% confidence intervals were computed from distributions of 30,000 bootstrapped development, *α* and mortality *δ* parameter estimates. Significance of pellet quality effects on survivorship was computed following the same methods described in Figure 4. Pellet quality comparisons of *C. maculatus* survivorship labeled with different letters within each stage of weevil development, are significantly different from the null difference of resampled *C. maculatus* survivorship, (*p* < .05)

Stage‐specific development times (*α* ^−1^) and survivorship (*S*) of *C. maculatus* were inferred from the number of individuals in a particular stage through time and the cumulative number of individuals that died in each stage using a stage‐structured model fitted to the time‐series data. The overall fits of the model to the data were good (Figure 3), indicating that the model captured the main fluxes of individuals as they developed. Moreover, the confidence intervals around the parameter estimates (*α* ^−1^ and *S*) (Figures 4 and 5) support the assertion that the inference is statistically well‐posed given the data (Nelson et al., 2004). We found no effect of pellet quality on development time in the first instar stage or in the pupal stage (Figure 4, ESM: Figure S1). Similarly, development time did not differ between 100% and 95% pellet quality treatments throughout development. However, beginning in the second instar, we found significant differences among the remaining pellet quality treatments in development time, where generally, development time increased with decreasing pellet quality (Figure 4, ESM: Figure S1). Initially, this difference was found only among the two pellet quality extremes (90% and 100%); however, as individuals developed into their third and fourth instar stages, we also found differences between 90% and 95% (Figure 4, ESM: Figure S1).

In contrast to development time, we found different stage survivorships among pellet quality treatments beginning in the first instar (Figure 5, ESM: Figure S2). The difference was initially only significant between the two pellet quality extremes (90% and 100%). However, in the second and third instars, we found different survivorships between 90% and 95% pellet qualities as well. Generally, within the first three instars, survivorship increased with increasing pellet quality. However, we found no differences between 90% and 95% in the first instar, nor did we find differences between 95% and 100% in the second or third instars. In the fourth instar, we found a different trend. Specifically, as in the first instar, we found no differences between 90% and 95%; however, instead of an increase in survivorship between 95% and 100%, we found a decrease and found no difference in fourth instar survivorship between 90% and 100% (Figure 5, ESM: Figure S2).

**Figure 6:**
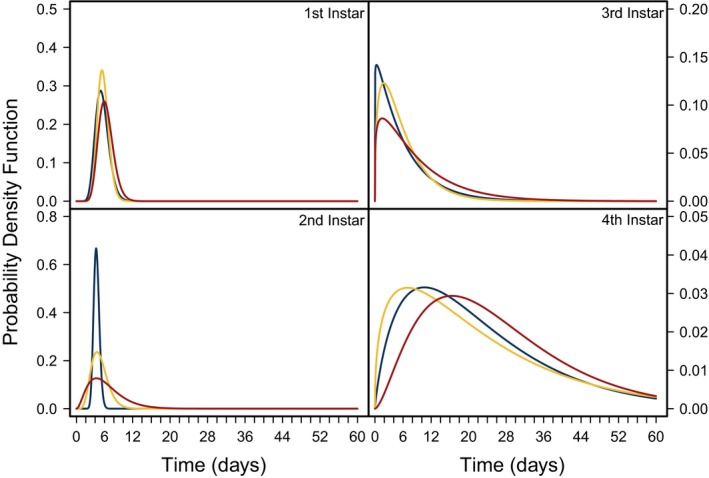
Probability density function estimating the length of time *C. maculatus* spends in each stage of development (1st, 2nd, 3rd, and 4th instars) for three pellet qualities (90% black‐eye pea: 10% filler, 95% black‐eye pea: 5% filler, and 100% black‐eye pea: 0% filler) in red, yellow, and blue lines, respectively. The length of time *C. maculatus* spent in each stage of development was estimated for each pellet quality from a Gamma density distribution function using the most likely parameter values, *α* and *k* as the scale and shape parameters in the function dgamma, respectively

To connect these results to potential consequences for the susceptibility of larvae to parasitism, we estimated the length of time larvae spent in each instar using the most likely parameter values *α* and *k* using dgamma (R Core Team, 2018), where *k* was fitted as the shape parameter, and *kα* was fitted as the scale parameter. As pellet quality decreased, variation in the length of time *C. maculatus* was estimated to spend in each instar stage of development increased (Figure 6). The variation across pellet quality treatments appears to be stage‐specific; however, the fourth instar showed a much wider distribution than other instars.

## 4 DISCUSSION

Food quality and availability are widely asserted to impact consumer growth and development (Awmack & Leather, 2002; Elser, Dobberfuhl, MacKay, & Schampel, 1996; Scriber & Slansky, 1981). As food quality decreases, growth and development are often impeded, resulting in smaller individuals (Berg & Merritt, 2009) that develop slower and have higher rates of mortality (Colasurdo, Gélinas, & Despland, 2009; Moreau, Benrey, & Thiery, 2006; Elser et al., 1996; Sterner, 1993). As expected, our results showed that head capsule and biomass growth of *C. maculatus* generally decreased with decreasing food quality (Figures 1 and 2), and development rates and survivorship decreased with decreasing food quality (Figures 4 and 5). While we found significant food quality effects on all larval life history traits, the size of these effects was not consistent across larval instars. Specifically, larvae in the 90% quality treatment spent 38% and 37% more time in the second and third instars, respectively, relative to larvae in the 100% quality treatment, but spent only 18% more time in the fourth instar. Similarly, larval dry mass was 34%, 53%, and 63% smaller in the 90% quality treatment than the 100% quality treatment in the second, third, and fourth instars, respectively, and larvae head capsules were 10%, 15%, and 12% smaller, respectively. This suggests that food quality affects larval size and that the effects of food quality on larval growth and development varies across developmental stages.

We generally found large effects of food quality on growth, development, and survivorship between food quality extremes (e.g., 90% vs. 100%) and small effects or no effects between similar food qualities (e.g., 90% vs. 95% and 95% vs. 100%). However, as animals grew and progressed through their instars, we found larger effects of pellet quality on growth and development between the two poorest pellet qualities (90% vs. 95%). This disparity in food quality effects on growth and development between earlier and later instars could be an artifact of the amount of food consumed, where the more food that is consumed, the more apparent the effects are on the consumer. For example, third and fourth instar larvae across taxa typically consume between 90% and 180%, respectively, more food than younger first and second instars (Barrigossi, Zimmermann, & Lima, 2002; Santos, Almeida, Castro‐Guedes, & Penteado, 2014), and therefore, the negative effects of consuming poor food quality might not be as evident in younger instar stages compared with older stages simply because they have not consumed enough of it.

Additionally, this magnified difference in food quality effects on growth and development between 90% and 95% pellet qualities in the third and fourth instars was not evident in the comparison between 95% and 100% pellet qualities. Instead, we saw no difference between 95% and 100% pellet qualities on growth in the third instar, although we did see a treatment difference in biomass growth in the fourth instar. This suggests that larvae consuming 95% pellet qualities may be compensating for lower quality food by consuming more of it. Compensatory growth in response to poor food quality has been demonstrated across a variety of taxa (Simpson & Simpson, 1989 for a review; Berner, Blanckenhorn, & Korner, 2005; Dmitriew & Rowe, 2005; Flores, Larranaga, & Elosegi, 2013), and it has also been shown that low‐quality food items have shorter gut transit times compared with high‐quality food items (Mitra & Flynn, 2007). Thus, with faster digestion and excretion rates of poor quality food resources, consumers may in turn increase their ingestion rates.

Unlike food quality effects on growth and development, food quality effects on larval survivorship appear to largely impact the first and second instars (Figure 5). This implies that larvae are more vulnerable to the effects of reduced food quality at younger developmental stages than older ones, which is consistent with the findings of Hódar, Zamora, and Castro (2002) and other studies across taxa (Waldbauer, 1968 for a review; Wikelski, Gall, & Trillmich, 1993 and Benavides, Cancino, & Ojeda, 1994 for examples in fish and reptiles, respectively), where it is suggested that food digestibility disproportionately impacts early larval survivorship. Since our pellet qualities were adjusted by using an indigestible filler, early instar larvae exposed to the lower pellet quality treatments in our study may have been less able to digest their food. Food digestibility may also explain why larvae consuming 95% pellets had lower biomass than their counterparts feeding on 100% pellets. Even if larvae consuming 95% pellets were compensating for lower food quality by consuming more food, their increased inability to digest their food may have prevented them from growing as large as larvae feeding on 100% pellets.

Interestingly, we found that survivorship in the fourth instar decreased in the highest food quality environment and was not significantly different from the lowest food quality treatment. This is consistent with the notion of a physiological cost to developing fast (Zera & Harshman, 2001), where animals that develop at a faster rate experience lower viability in later stages (Blanckenhorn, 1998; Chippindale, Alipaz, Chen, & Rose, 1997).

**Figure 7:**
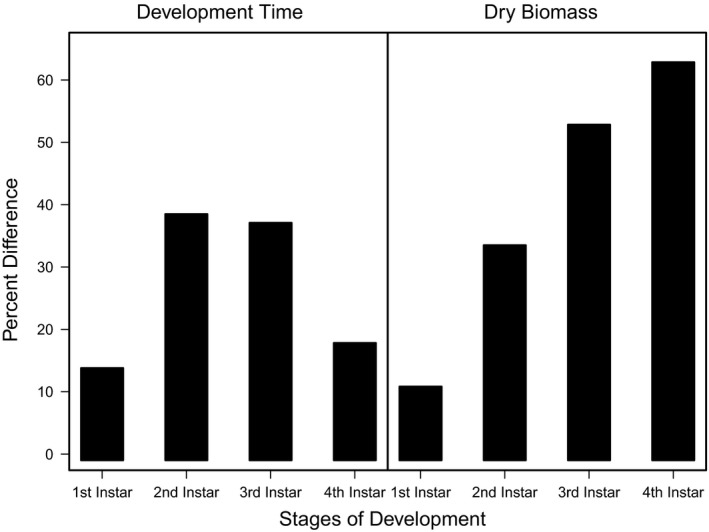
Percent differences of *Callosobruchus maculatus* development time and dry biomass in each instar stage of development (*L*1, *L*2, *L*3, and *L*4) comparing two pellet qualities (90% black‐eye pea: 10% filler and 100% black‐eye pea: 0% filler)

Overall, our results show that the effects of food quality on juvenile life histories vary across instars. Since pellet quality influences growth and development differentially across instars, we can then infer that any changes in food quality may have implications for a larva's instar‐specific interactions with its environment. For example, Ngamo et al. (2007) showed that *C. maculatus* is most vulnerable to its generalist parasitoid *Anisopteromalus calandrae* during its fourth instar. We found that *C. maculatus* larvae feeding on poor food quality on average require 4.6 days longer to progress through their fourth instar compared with larvae feeding on higher food qualities, are 60% lighter, and have 7% lower probability of surviving until their fourth instar (Figure 7). Thus, *C. maculatus* feeding on low‐quality food will spend more time in this vulnerable stage and would be exposed to its parasitoid *A. calandrae* for longer time, potentially increasing its susceptibility to parasitism.

Furthermore, we found that the impact of food quality on larval life history traits generates a trade‐off between the larval size and the development time in the vulnerable stage of *C. maculatus* that will likely scale‐up to impact its susceptibility to being parasitized due to parasitoid fitness being positively correlated with host size (Wang, Yang, Wu, & Gould, 2008). If larvae feeding on poorer quality food are more vulnerable to their parasitoids due to longer exposure times, then the size of the larval community will likely be impacted, which in turn can impact parasitoid fitness and parasitoid dynamics (Bukovinszky, Van Veen, Jongema, & Dicke, 2008). Alternatively, fourth instar larvae feeding on poor food quality may not be large enough to support the growth and development of parasitoids' offspring. Since larval size is an important predictor of parasitoid success and fitness (Hardy, Griffiths, & Godfray, 1992; Wang et al., 2008), smaller larvae exposed for longer periods may not be as susceptible to parasitoids as larger larvae exposed for shorter periods.

The trade‐off between larval size and development time in response to food quality could have implications for larval competitive ability. Age‐ and size‐structure often impact larval competitive ability, where older, larger larvae outcompete younger, smaller larvae, both intraspecifically and interspecifically (Cameron, Wearing, Rohani, & Sait, 2006). Our results show food quality primarily effects differences in body size in the third and fourth larval instars with little differences in the first and second larval instars. Thus, we expect food quality will impact the strength of asymmetric competitive ability because the disparity in size structure is largely increased across a food quality gradient for older instars, where poorer food qualities produce smaller, older larvae. Our results also show food quality effects differences in larval development time, where poorer food quality delays larval development. Delays in larval development may lower an individual's exploitative competitive ability since the first larvae to reach reproductive maturity, regardless of size, have access to mates and shared resources sooner than individuals developing slowly on poor food resources (reviewed in Metcalfe & Monaghan, 2003).

The net effect of larval life history traits in response to food quality on the stage‐specific interactions larvae often face throughout their juvenile environment remains to be seen. However, by conducting detailed instar‐specific studies, it is possible to layout the potential processes for how larval food resources may impact an individual's competitive ability, its susceptibility to parasitism and predation, as well as scale through this type of food chain to affect parasitoid and predator fitness.

## CONFLICT OF INTEREST

None declared.

## AUTHOR CONTRIBUTION

LH, WN, and SL conceived the ideas and designed methodology; LH executed the experiment and collected the data; LH and WN led the writing of the manuscript and analyzed the data. All authors contributed critically to the drafts and gave final approval for publication.

## Data Availability

Data available from the Dryad Digital Repository https://doi.org/10.5061/dryad.d7wm37px7 (Holmes, Nelson, & Lougheed, 2019).
