# Supplementary material for: Food quality effects on instar‐specific life histories of a holometabolous insect
Source: Ecol Evol. 2020 Jan 3;10(2):626–37. doi: 10.1002/ece3.5790 (PMC6988550; doi:10.1002/ece3.5790)
Supplement: Supplementary file 1 [file ECE3-10-626-s001.pdf]

Electronic Supplementary Material: Holmes, L. A., Nelson, W. A., & Lougheed, S. C.

Table S1 – Computed dispersion parameters for the observed number of living *Callosobruchus maculatus* in each stage and the number of dead *C. maculatus* in all stages for each food quality treatment. Indices of dispersion were computed using the ratio of the residual deviance to the degrees of freedom deviance. Using these indices of dispersion as the scale parameter value in the function *rTweedie* from the package *mgcv* (Wood, 2017), we resampled our observed data by generating Tweedie random deviates which were used to generate distributions of most likely parameter estimates for each parameter, development time, α^{-1} and mortality rate, δ.

**Living weevils in each stage of development**	**Dispersion Parameter**		
	90% black-eye pea:10% filler	95% black-eye pea:5% filler	100% black-eye pea:0% filler
1st Larval Instar	0.11	0.23	0.11
2nd Larval Instar	0.48	0.71	1.14
3rd Larval Instar	3.14	1.72	0.94
4th Larval Instar	0.83	1.26	0.52
5th Larval Instar	0.73	1.61	2.45
Adults	0.48	0.46	0.72
**Dead weevils in all stages of development**	0.0259	0.0002	0.0025

Table S2 – Simultaneous tests for general linear hypotheses. Multiple comparisons of means: Tukey Contrasts. Fit glm(head capsule width ~ pellet quality * instar stage, family = poisson(link = ‘log’, adjusted for Bonferroni correction)

**Comparisons**	**Estimate**	**Std. Error**	**z value**	**Pr(>|z|)**
95.L1 - 90.L1	0.0042	0.0225	0.1845	1
100.L1 - 90.L1	-0.0069	0.0226	-0.3055	1
90.L2 - 90.L1	0.3889	0.0228	17.0470	< 0.001

95.L2 - 90.L1	0.4521	0.0249	18.1513	< 0.001
100.L2 - 90.L1	0.4971	0.0254	19.6004	< 0.001
90.L3 - 90.L1	0.7871	0.0204	38.5325	< 0.001
95.L3 - 90.L1	0.9019	0.0219	41.2275	< 0.001
100.L3 - 90.L1	0.9488	0.0216	43.9800	< 0.001
90.L4 - 90.L1	1.2626	0.0174	72.5973	< 0.001
95.L4 - 90.L1	1.3515	0.0171	78.9687	< 0.001
100.L4 - 90.L1	1.3836	0.0171	80.8173	< 0.001
100.L1 - 95.L1	-0.0111	0.0231	-0.4790	1
90.L2 - 95.L1	0.3848	0.0233	16.5182	< 0.001
95.L2 - 95.L1	0.4479	0.0253	17.6728	< 0.001
100.L2 - 95.L1	0.4930	0.0258	19.1115	< 0.001
90.L3 - 95.L1	0.7829	0.0210	37.3536	< 0.001
95.L3 - 95.L1	0.8977	0.0224	40.1226	< 0.001
100.L3 - 95.L1	0.9447	0.0221	42.7845	< 0.001
90.L4 - 95.L1	1.2585	0.0180	69.8541	< 0.001
95.L4 - 95.L1	1.3473	0.0177	75.9167	< 0.001
100.L4 - 95.L1	1.3795	0.0178	77.7014	< 0.001
90.L2 - 100.L1	0.3958	0.0234	16.9278	< 0.001
95.L2 - 100.L1	0.4590	0.0254	18.0504	< 0.001
100.L2 - 100.L1	0.5041	0.0259	19.4790	< 0.001
90.L3 - 100.L1	0.7940	0.0211	37.7018	< 0.001
95.L3 - 100.L1	0.9088	0.0225	40.4478	< 0.001
100.L3 - 100.L1	0.9558	0.0222	43.1005	< 0.001
90.L4 - 100.L1	1.2695	0.0181	70.0169	< 0.001
95.L4 - 100.L1	1.3584	0.0179	76.0352	< 0.001
100.L4 - 100.L1	1.3906	0.0179	77.8083	< 0.001
95.L2 - 90.L2	0.0632	0.0256	2.4671	0.8990
100.L2 - 90.L2	0.1082	0.0261	4.1547	0.0022
90.L3 - 90.L2	0.3982	0.0213	18.7156	< 0.001
95.L3 - 90.L2	0.5130	0.0227	22.6279	< 0.001
100.L3 - 90.L2	0.5599	0.0224	25.0200	< 0.001
90.L4 - 90.L2	0.8737	0.0184	47.5325	< 0.001
95.L4 - 90.L2	0.9626	0.0181	53.1264	< 0.001
100.L4 - 90.L2	0.9947	0.0181	54.8827	< 0.001
100.L2 - 95.L2	0.0451	0.0279	1.6151	1
90.L3 - 95.L2	0.3350	0.0235	14.2524	< 0.001
95.L3 - 95.L2	0.4498	0.0248	18.1557	< 0.001
100.L3 - 95.L2	0.4968	0.0245	20.2684	< 0.001
90.L4 - 95.L2	0.8105	0.0209	38.7410	< 0.001
95.L4 - 95.L2	0.8994	0.0207	43.4673	< 0.001
100.L4 - 95.L2	0.9316	0.0207	45.0097	< 0.001
90.L3 - 100.L2	0.2899	0.0240	12.0860	< 0.001

95.L3 - 100.L2	0.4047	0.0252	16.0388	< 0.001
100.L3 - 100.L2	0.4517	0.0250	18.0865	< 0.001
90.L4 - 100.L2	0.7655	0.0215	35.6615	< 0.001
95.L4 - 100.L2	0.8543	0.0212	40.2223	< 0.001
100.L4 - 100.L2	0.8865	0.0212	41.7260	< 0.001
95.L3 - 90.L3	0.1148	0.0203	5.6649	< 0.001
100.L3 - 90.L3	0.1618	0.0199	8.1121	< 0.001
90.L4 - 90.L3	0.4755	0.0153	31.0438	< 0.001
95.L4 - 90.L3	0.5644	0.0150	37.6219	< 0.001
100.L4 - 90.L3	0.5966	0.0150	39.7459	< 0.001
100.L3 - 95.L3	0.0470	0.0214	2.1918	1
90.L4 - 95.L3	0.3607	0.0172	20.9684	< 0.001
95.L4 - 95.L3	0.4496	0.0169	26.5680	< 0.001
100.L4 - 95.L3	0.4818	0.0169	28.4572	< 0.001
90.L4 - 100.L3	0.3138	0.0168	18.6567	< 0.001
95.L4 - 100.L3	0.4026	0.0165	24.3570	< 0.001
100.L4 - 100.L3	0.4348	0.0165	26.2916	< 0.001
95.L4 - 90.L4	0.0889	0.0105	8.4615	< 0.001
100.L4 - 90.L4	0.1210	0.0105	11.5117	< 0.001
100.L4 - 95.L4	0.0322	0.0100	3.2006	0.0905

Table S3 – Simultaneous tests for general linear hypotheses. Multiple comparisons of means: Tukey Contrasts. Fit glm(Dry mass ~ pellet quality * instar stage, family = Gamma(link = ‘inverse’, adjusted for Bonferroni correction)

**Comparisons**	**Estimate**	**Std. Error**	**z value**	**Pr(>|z|)**
95.L1 - 90.L1	-9.4647	11.338	-0.8348	1
100.L1 - 90.L1	-21.8421	11.0728	-1.9726	1
90.L2 - 90.L1	-127.2863	8.2794	-15.3738	< 0.001
95.L2 - 90.L1	-136.6352	8.2158	-16.6308	< 0.001
100.L2 - 90.L1	-140.2813	8.1921	-17.1241	< 0.001
90.L3 - 90.L1	-163.7071	7.9759	-20.5252	< 0.001
95.L3 - 90.L1	-166.974	7.9717	-20.9457	< 0.001
100.L3 - 90.L1	-167.4116	7.9709	-21.0028	< 0.001
90.L4 - 90.L1	-171.0742	7.9664	-21.4745	< 0.001
95.L4 - 90.L1	-171.6435	7.9663	-21.5462	< 0.001
100.L4 - 90.L1	-171.7895	7.9663	-21.5646	< 0.001
100.L1 - 95.L1	-12.3774	11.1461	-1.1105	1
90.L2 - 95.L1	-117.8215	8.3772	-14.0646	< 0.001
95.L2 - 95.L1	-127.1705	8.3143	-15.2954	< 0.001

100.L2 - 95.L1	-130.8166	8.2909	-15.7784	< 0.001
90.L3 - 95.L1	-154.2424	8.0774	-19.0956	< 0.001
95.L3 - 95.L1	-157.5092	8.0732	-19.5101	< 0.001
100.L3 - 95.L1	-157.9469	8.0724	-19.5663	< 0.001
90.L4 - 95.L1	-161.6095	8.0679	-20.0311	< 0.001
95.L4 - 95.L1	-162.1788	8.0679	-20.1019	< 0.001
100.L4 - 95.L1	-162.3247	8.0678	-20.12	< 0.001
90.L2 - 100.L1	-105.4442	8.0145	-13.1566	< 0.001
95.L2 - 100.L1	-114.7931	7.9488	-14.4416	< 0.001
100.L2 - 100.L1	-118.4392	7.9243	-14.9464	< 0.001
90.L3 - 100.L1	-141.865	7.7006	-18.4225	< 0.001
95.L3 - 100.L1	-145.1318	7.6963	-18.8574	< 0.001
100.L3 - 100.L1	-145.5695	7.6954	-18.9164	< 0.001
90.L4 - 100.L1	-149.2321	7.6907	-19.4041	< 0.001
95.L4 - 100.L1	-149.8014	7.6906	-19.4784	< 0.001
100.L4 - 100.L1	-149.9474	7.6906	-19.4974	< 0.001
95.L2 - 90.L2	-9.349	3.0209	-3.0948	0.13
100.L2 - 90.L2	-12.9951	2.9558	-4.3965	< 0.001
90.L3 - 90.L2	-36.4208	2.2896	-15.9073	< 0.001
95.L3 - 90.L2	-39.6877	2.2749	-17.4456	< 0.001
100.L3 - 90.L2	-40.1253	2.272	-17.6604	< 0.001
90.L4 - 90.L2	-43.7879	2.2561	-19.4086	< 0.001
95.L4 - 90.L2	-44.3572	2.2558	-19.6637	< 0.001
100.L4 - 90.L2	-44.5032	2.2557	-19.7288	< 0.001
100.L2 - 95.L2	-3.6461	2.7725	-1.3151	1
90.L3 - 95.L2	-27.0718	2.0475	-13.2218	< 0.001
95.L3 - 95.L2	-30.3387	2.0311	-14.9367	< 0.001
100.L3 - 95.L2	-30.7763	2.0279	-15.1764	< 0.001
90.L4 - 95.L2	-34.4389	2.01	-17.1335	< 0.001
95.L4 - 95.L2	-35.0082	2.0097	-17.4199	< 0.001
100.L4 - 95.L2	-35.1542	2.0096	-17.4929	< 0.001
90.L3 - 100.L2	-23.4257	1.9501	-12.0123	< 0.001
95.L3 - 100.L2	-26.6926	1.933	-13.8092	< 0.001
100.L3 - 100.L2	-27.1302	1.9296	-14.0604	< 0.001
90.L4 - 100.L2	-30.7929	1.9108	-16.1155	< 0.001
95.L4 - 100.L2	-31.3622	1.9104	-16.4167	< 0.001
100.L4 - 100.L2	-31.5081	1.9103	-16.4936	< 0.001
95.L3 - 90.L3	-3.2669	0.4918	-6.6426	< 0.001
100.L3 - 90.L3	-3.7045	0.4782	-7.7461	< 0.001
90.L4 - 90.L3	-7.3671	0.3957	-18.6187	< 0.001
95.L4 - 90.L3	-7.9364	0.3938	-20.1523	< 0.001
100.L4 - 90.L3	-8.0824	0.3936	-20.5359	< 0.001
100.L3 - 95.L3	-0.4376	0.4024	-1.0874	1

90.L4 - 95.L3	-4.1002	0.2997	-13.682	< 0.001
95.L4 - 95.L3	-4.6695	0.2972	-15.7108	< 0.001
100.L4 - 95.L3	-4.8155	0.2969	-16.22	< 0.001
90.L4 - 100.L3	-3.6626	0.2769	-13.2293	< 0.001
95.L4 - 100.L3	-4.2319	0.2742	-15.4343	< 0.001
100.L4 - 100.L3	-4.3779	0.2738	-15.9876	< 0.001
95.L4 - 90.L4	-0.5693	0.0551	-10.3368	< 0.001
100.L4 - 90.L4	-0.7153	0.0533	-13.4299	< 0.001
100.L4 - 95.L4	-0.146	0.037	-3.9483	< 0.005

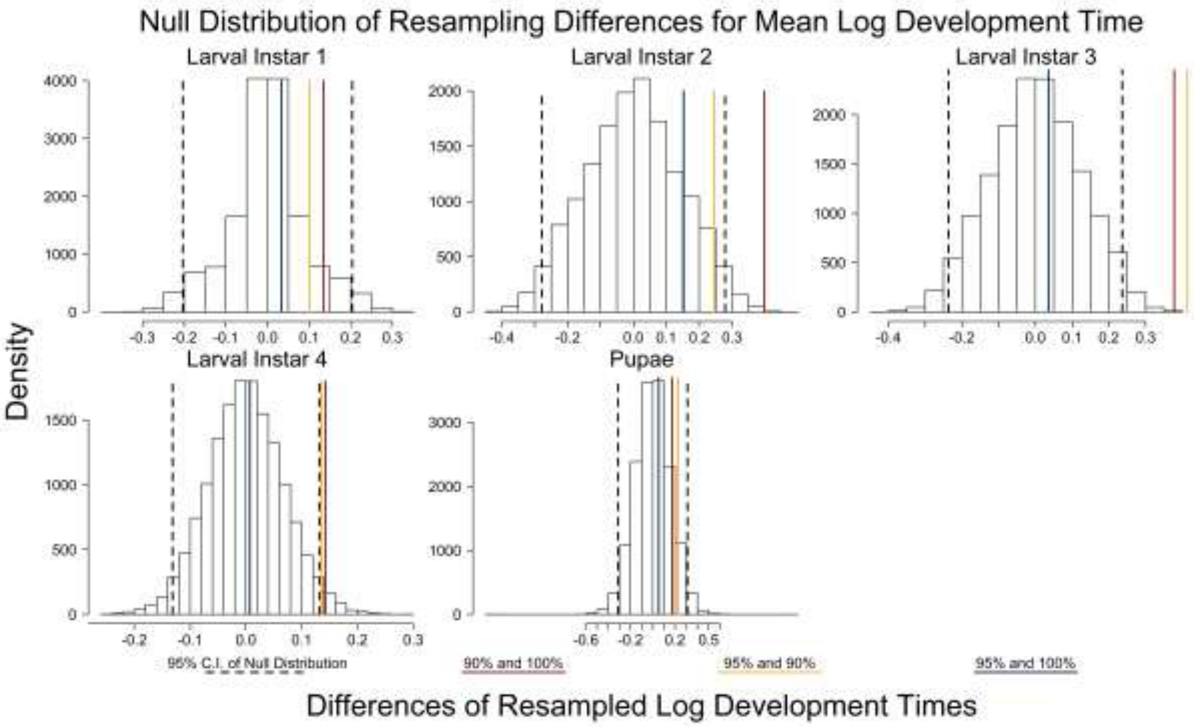

Figure S1 – Frequency null distribution of resampling differences of *Callosobruchus maculatus* development time, α^{-1} across food quality treatments for each weevil stage of development (L1, L2, L3, L4, L5(Pupae), and Adults) with 95% confidence intervals plotted (dashed lines). Observed food quality differences in development time, α^{-1} are plotted for 90%-95%, 95%-100% and 90%-100% comparisons in blue, grey and red, respectively. Any observed treatment comparison line located outside of the null distributions' 95% confidence intervals are significantly different from the null, ($p < 0.05$).

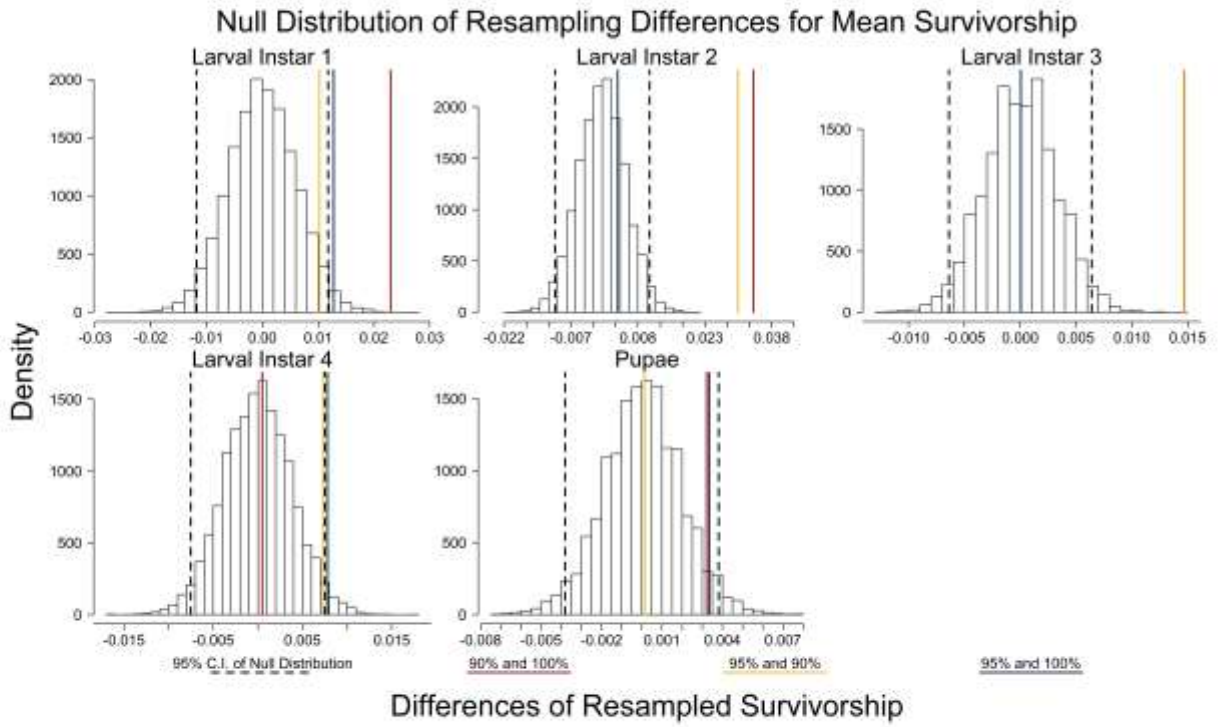

Figure S2 – Frequency null distribution of resampling differences of *Callosobruchus maculatus* survivorship, ($e^{-\delta\alpha^{-1}}$), across food quality treatments for each weevil stage of development (L1, L2, L3, L4, L5(Pupae), and Adults) with 95% confidence intervals plotted (dashed lines), where α and δ are development and mortality rates estimated from the instar-structured model (Equation 1 in the manuscript) fit to our time series data (Fig. 3 in the manuscript). Observed food quality differences in survivorship are plotted for 90%-95%, 95%-100% and 90%-100% comparisons. Any observed treatment comparison line located outside of the null distributions' 95% confidence intervals are significantly different from the null, ($p < 0.05$).
